# Supplementary material for: Atlas of proteomic signatures of brain structure and its links to brain disorders
Source: Nat Commun. 2025 Jun 2;16:5092. doi: 10.1038/s41467-025-60185-7 (PMC12130460; doi:10.1038/s41467-025-60185-7)
Supplement: Supplementary file 1 — Supplementary Information [file 41467_2025_60185_MOESM1_ESM.pdf]

Atlas of Proteomic Signatures of Brain Structure and Its Links to Brain Disorders

Supplementary Material

Supplementary Methods

Supplementary Figures

Figure S1 Flowchart of participant inclusion and exclusion.

Figure S2 Gene Ontology (GO) enrichment of associated proteins from all the five structural measures.

Figure S3 Tissue enrichment of positively and negatively associated proteins for volume and mean diffusivity (MD) measures.

Figure S4 Tissue enrichment of positively and negatively associated proteins for area and thickness measures.

Figure S5 The causal effect of protein on brain structure in the forward MR at strict clumping *P* threshold.

Figure S6 The causal effect of protein on disease in the forward MR at strict clumping *P* threshold.

Figure S7 The causal effect of brain structure on protein in the reverse MR.

Figure S8 The causal effect of disease on protein in the reverse MR.

Figure S9 The expression of coding genes across tissues for proteins exhibiting mediation effect.

Supplementary Methods

Preprocessing of proteomic data

The information regarding how the quality control was undertaken and how the protein data was normalized have been thoroughly documented in the Supplementary Information in previous publication[1], as well as on UK Biobank (UKB) official website (https://biobank.ndph.ox.ac.uk/showcase/refer.cgi?id=4658). The counts of known sequences were converted into Normalized Protein eXpression (NPX) values, which were derived through within-batch and across-batch normalization, using Olink's MyData Cloud Software. Within batch normalization centers data at NPX=0 by subtracting the plate-specific median per assay from all samples and assays in the same plate. Across-batch normalization calculates adjustment factors by determining the difference in assay-specific median NPX values for each batch. This process involves two steps: the first addresses plate-to-plate variation within a batch, while the second accounts for batch-to-batch variation across the study. Both steps involve shifting by an assay-specific fixed factor on the NPX scale: the plate median in the first step and the difference between assay-specific medians across batches in the second step.

The Olink workflow includes a inbuilt quality control system consisting of three engineered internal controls that are spiked into every sample and each abundance block. Olink's internal quality control (QC) assessment is performed at two levels; run QC and sample QC. For run QC, each abundance block per panel and sample plate should fulfil the mean absolute deviation (MAD) in both internal controls (Inc Ctrl and Amp Ctrl) which should not exceed 0.3 NPX, the deviation of sample QC level is allowed for up to 1/6 samples and in each panel the median of 90% assays in plate and negative controls should be in the accepted range from predefined values set during validation. The sample QC evaluates each sample individually using the internal controls (Inc Ctrl and Amp Ctrl), which should fall within ±0.3 NPX of the plate median across the abundance block. Additionally, the mean assay count for a sample must not be less than 500 counts. Samples that do not meet these criteria will receive a warning for the corresponding abundance block in the dataset.

Outliers were identified using two approaches applied to each protein panel: (1) principal component analysis (PCA), and (2) examining the median and interquartile range (IQR) of NPX across proteins by sample. Data points were removed if (1) a standardized PC1 (the component that captures the most variation) or PC2 (second largest component) value more than 5 standard deviations from the mean (which is zero in standardized PCA), or (2) a median NPX greater than 5 standard deviations from the mean median, or an IQR of NPX greater than 5 standard deviations from the mean IQR. We excluded outliers, data points with a QC or assay warning, and likely sample swaps, removing the sample across all panels if half or more of the panels were affected; the remaining data contained 56,695 samples and 52,790 individuals. Suspected sample swaps were identified by examining discrepancies between the proteomic-predicted sex and outliers from cis protein quantitative trait loci (pQTLs), where the standardized squared residuals for all proteins were summed for each individual and divided by the sum of squared protein levels. Samples with incorrect genotypes were expected to show larger values than those with correct genotypes.

Supplementary Figures

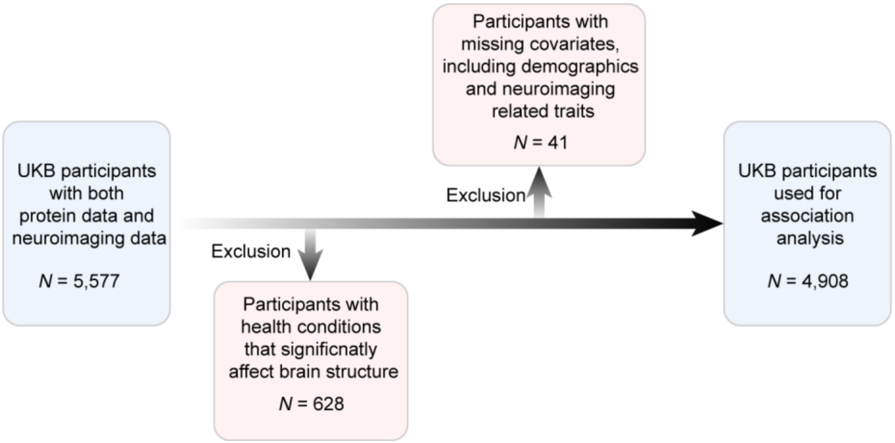

Figure S1 Flowchart of participant inclusion and exclusion. The details of inclusion and exclusion criteria for association analysis between proteins and brain structures, with the number of volume measure shown as the example.

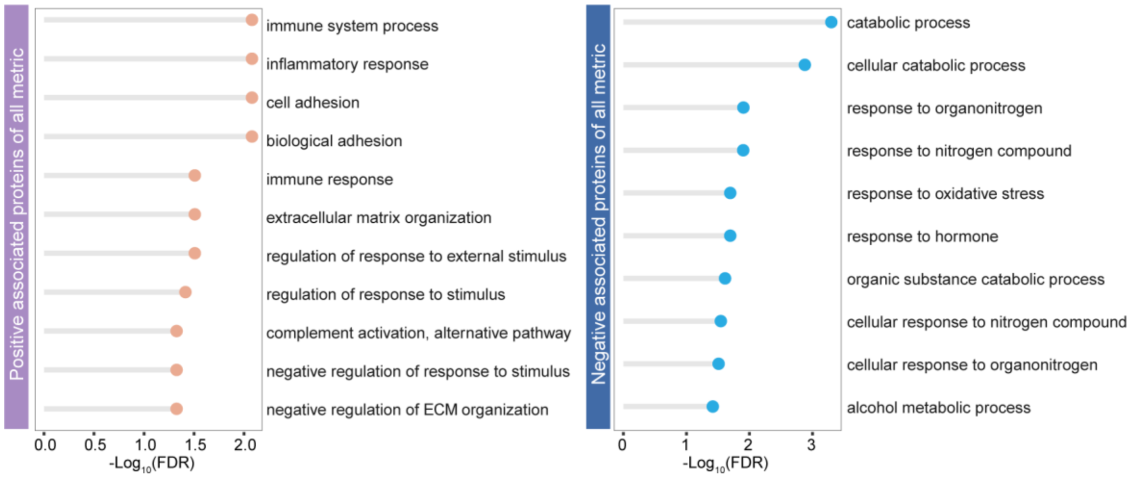

Figure S2 Gene Ontology (GO) enrichment of associated proteins from all the five structural measures. Positively and negatively associated proteins were enriched separately. Positively associated proteins were significantly enriched in biological processes such as immune system process, inflammatory response, and cell adhesion (left panel). Negatively associated proteins were significantly enriched in biological processes such as catabolic process, cellular catabolic process, and response to organonitrogen (right panel). The 2,920 proteins were used as the background of enrichment. An FDR-corrected $P < 0.05$ was considered significant. Source data are provided as a Source Data file.

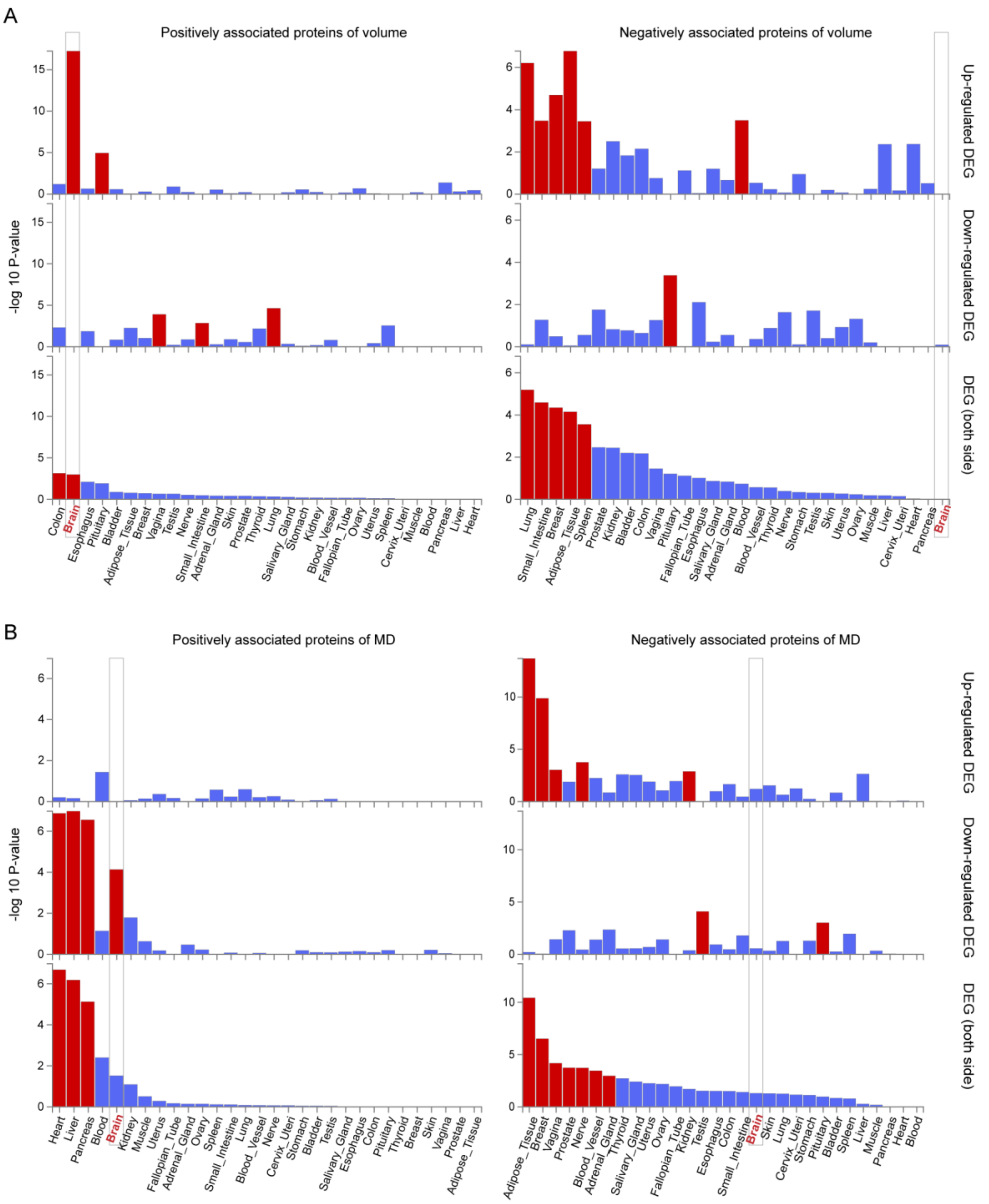

Figure S3 Tissue enrichment of positively and negatively associated proteins for volume and mean diffusivity (MD) measures. (A) Tissue enrichment results for proteins positively and negatively associated with brain volume. (B) Tissue enrichment results for proteins positively and negatively associated with MD measures. The 2,920 proteins were used as the background of enrichment. Significant enrichment at FDR-corrected $P < 0.05$ are colored in red. The brain tissue is also highlighted with rectangle and red color. Source data are provided as a Source Data file.

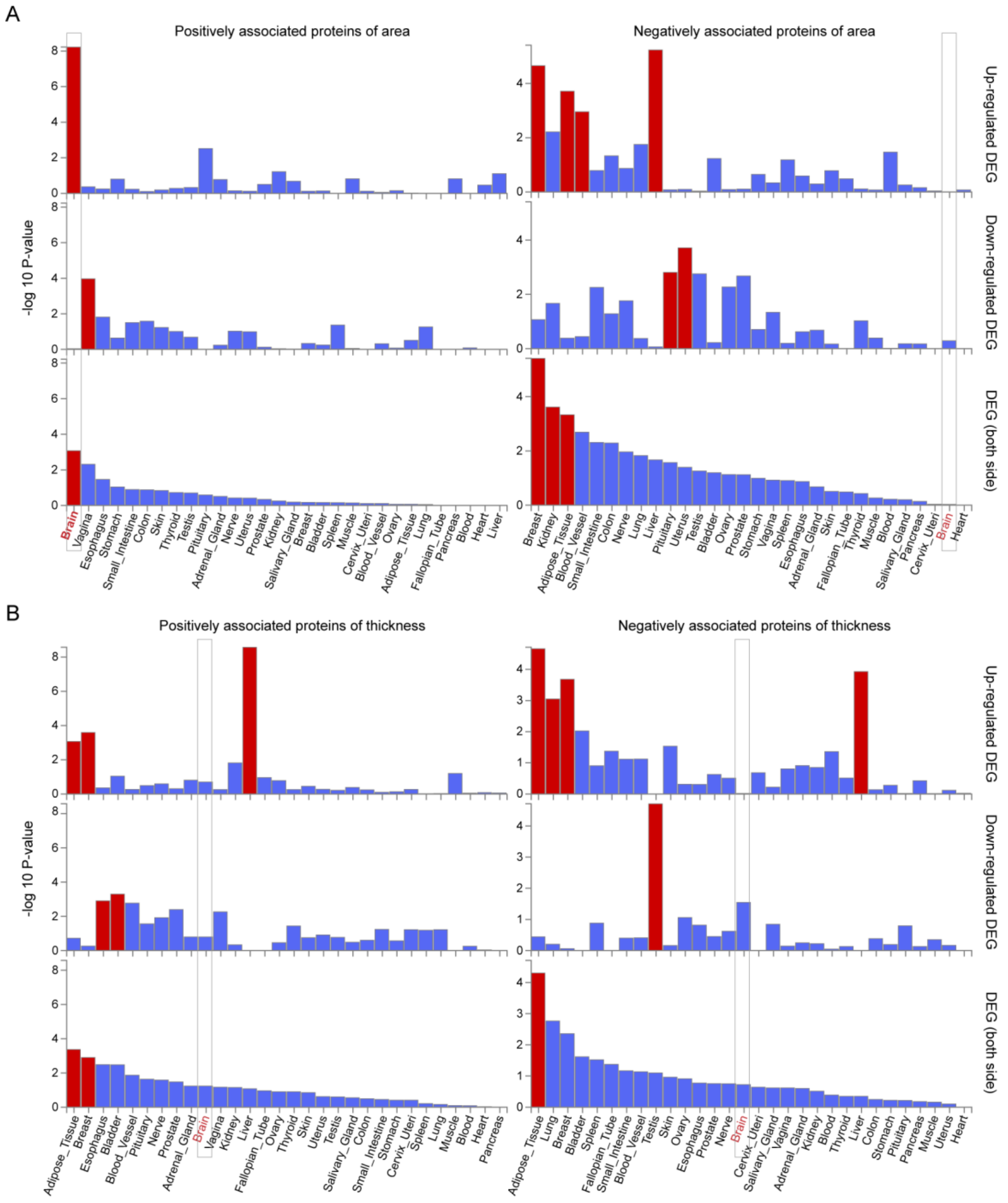

Figure S4 Tissue enrichment of positively and negatively associated proteins for area and thickness measures. (A) Tissue enrichment results for proteins positively and negatively associated with area. (B) Tissue enrichment results for proteins positively and negatively associated with thickness. The 2,920 proteins were used as the background of enrichment. Significant enrichment at FDR-corrected $P < 0.05$ are colored in red. The brain tissue is also highlighted with rectangle and red color. Source data are provided as a Source Data file

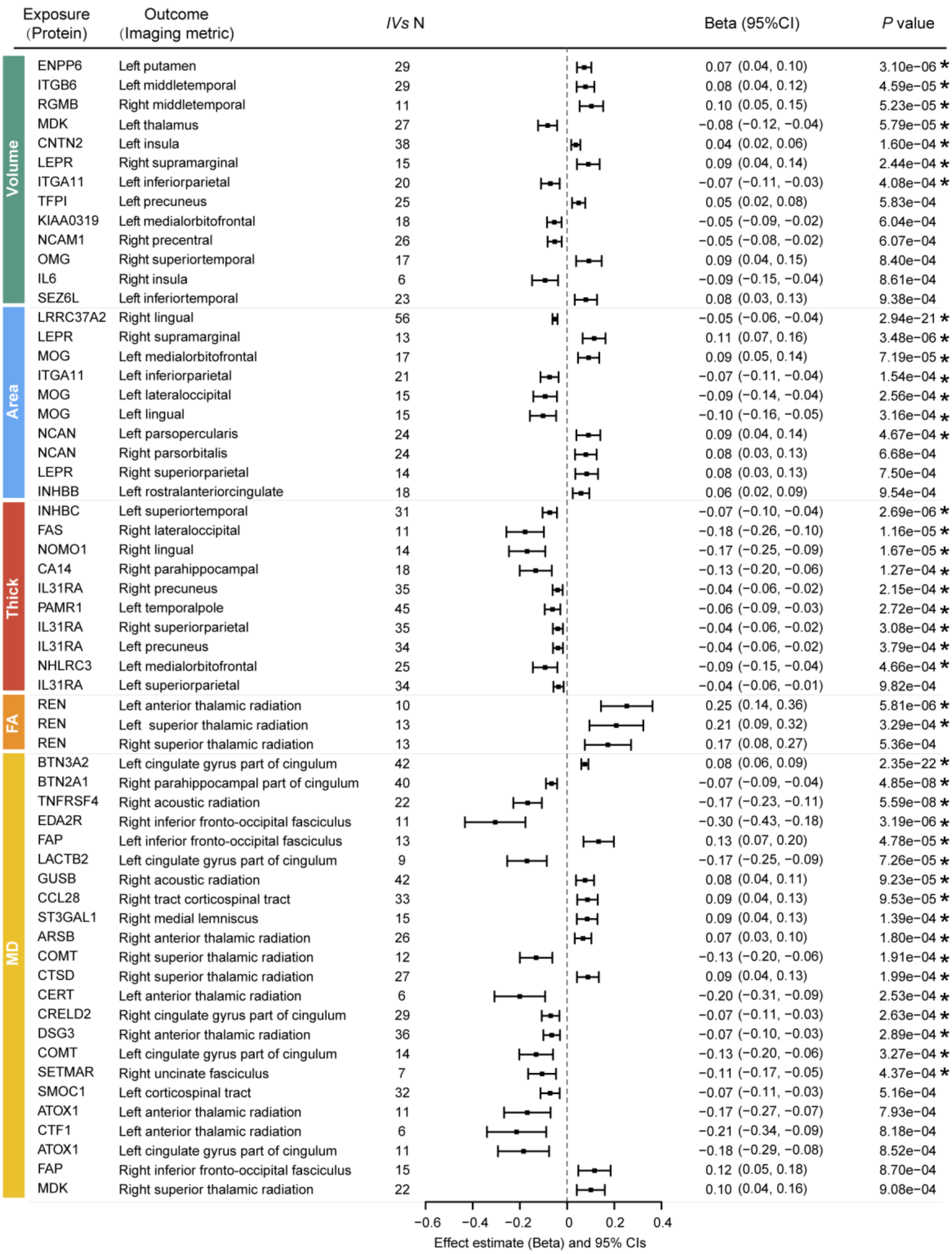

Figure S5 The causal effect of protein on brain structure in the forward mendelian randomization (MR) at a strict clumping *P* threshold. The forest plot shows the significant MR relationships of IVW method with a strict clumping *P* threshold of $5{\times}10^{-8}$. All MR results with a nominal $P < 0.001$ are shown. Raw *P* values are shown in the right most column. The MR relationships meet the significance threshold of FDR-corrected P<0.05 are marked with arterisk. All statistical tests were two-sided. Source data are provided as a Source Data file

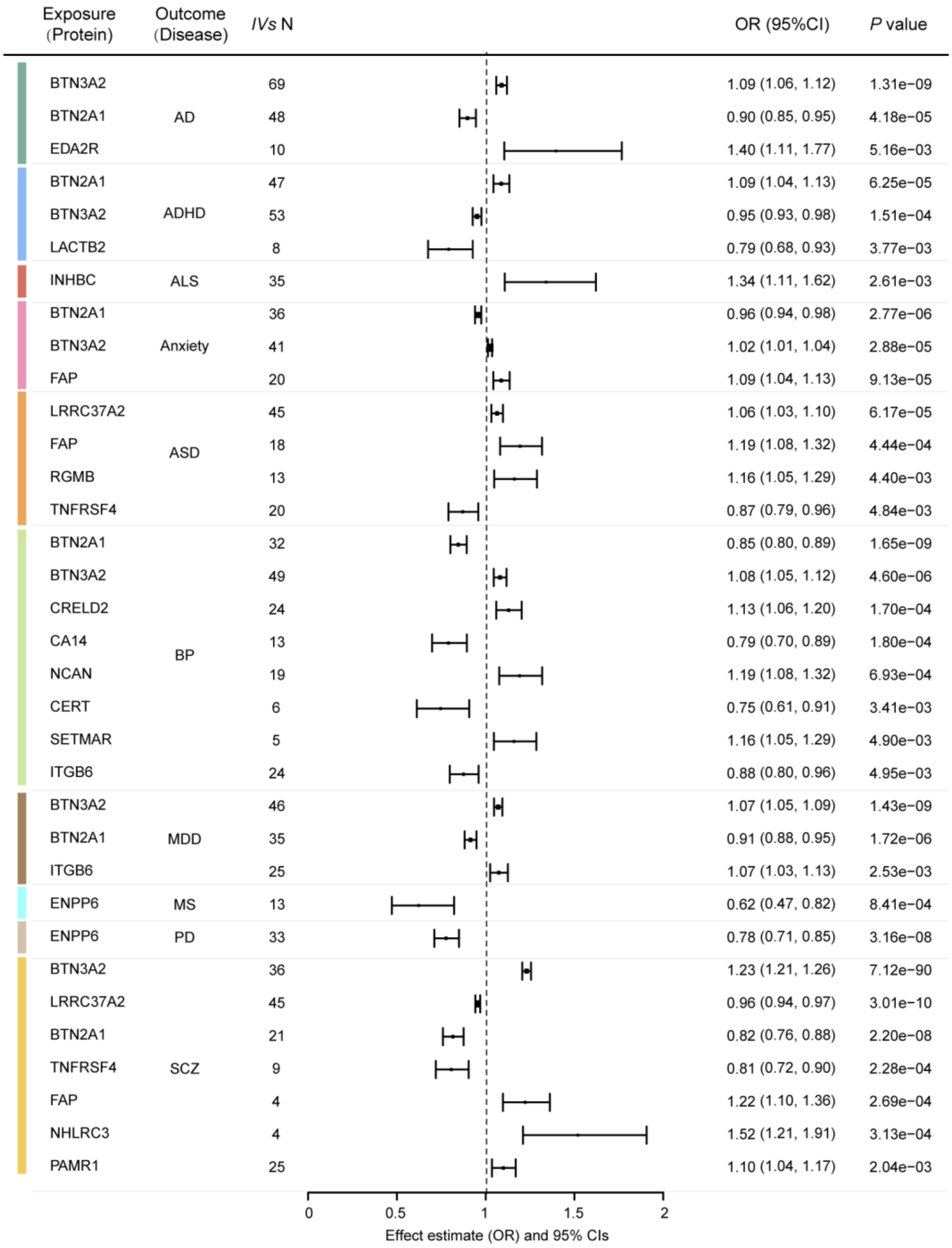

Figure S6 The causal effect of protein on disease in the forward MR at a strict clumping *P* threshold. The forest plot shows the significant MR relationships of IVW method with a strict clumping *P* threshold of 5×10^{-8}. Raw *P* values are shown in the right most column. The MR relationships meet the significance threshold of FDR-corrected $P<0.05$ are shown. All statistical tests were two-sided. Source data are provided as a Source Data file.

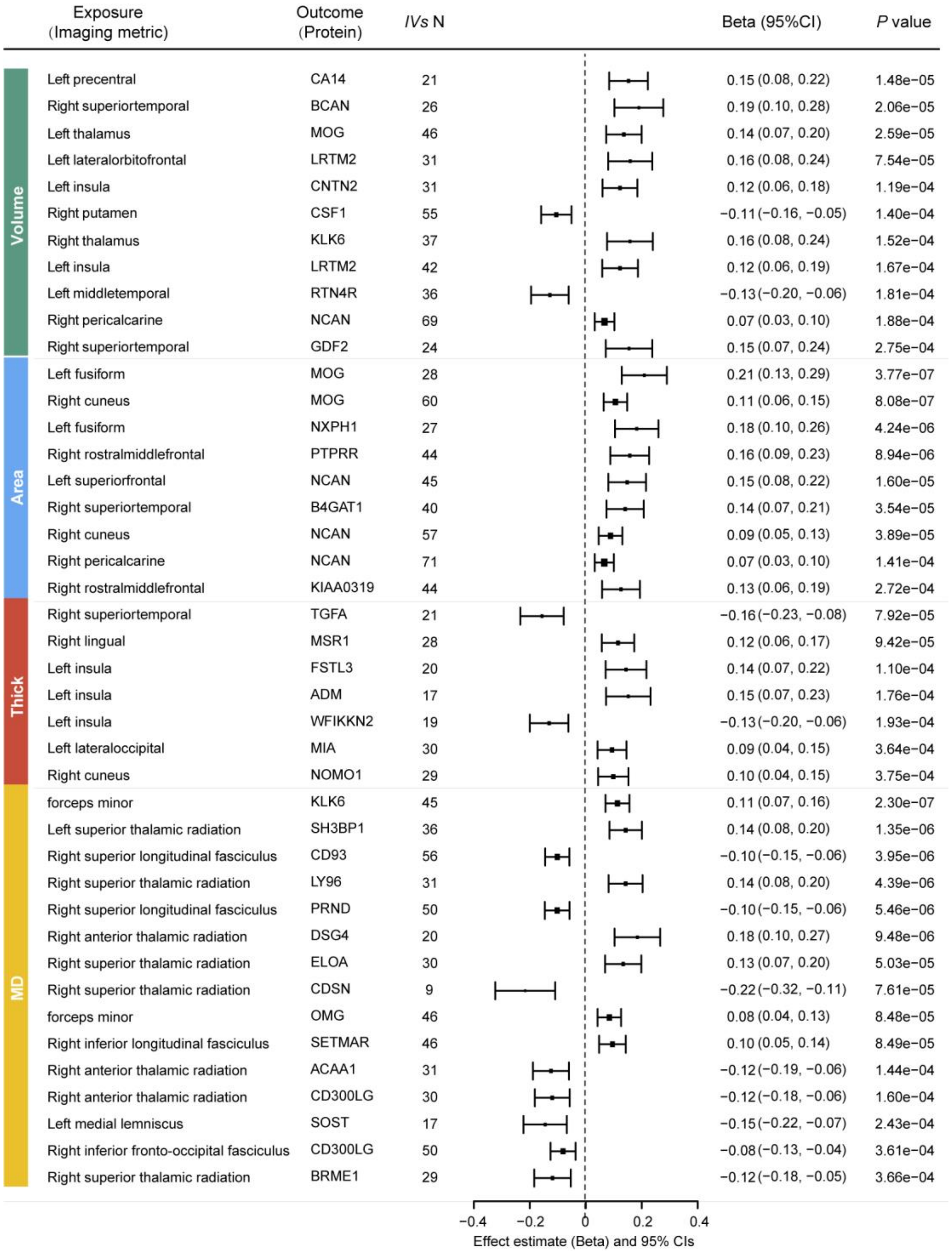

Figure S7 The causal effect of brain structure on protein. The forest plot shows the significant MR relationships of IVW method with a clumping *P* threshold of 5×10^{-6}. Raw *P* values are shown in the right most column. The MR relationships meet the significance threshold of FDR-corrected $P<0.05$ are shown. All statistical tests were two-sided. Source data are provided as a Source Data file.

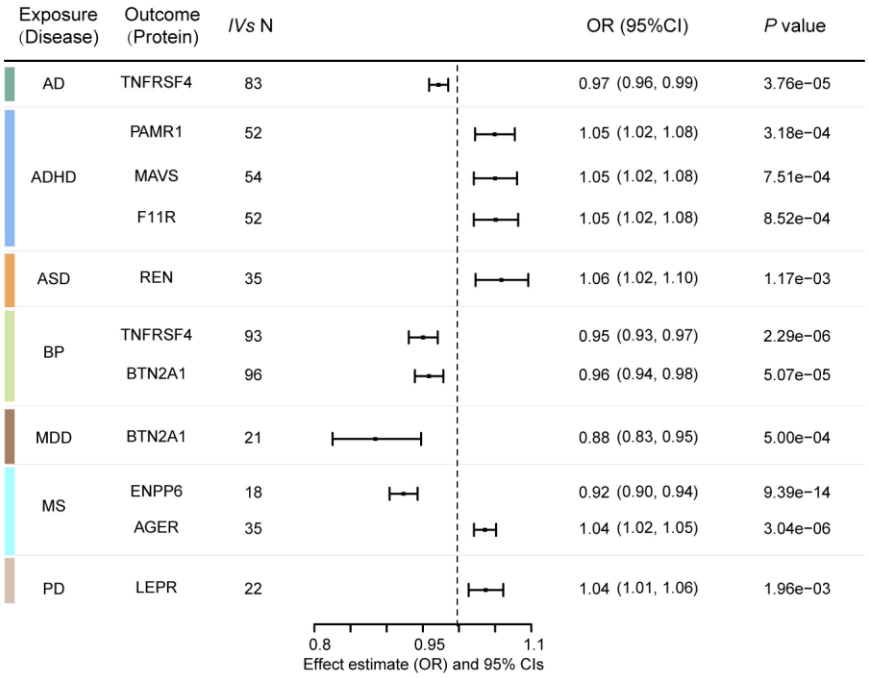

Figure S8 The causal effect of disease on protein in the reverse MR. The forest plot shows the significant MR relationships of IVW method with a clumping *P* threshold of 5×10^{-6}. Raw *P* values are shown in the right most column. The MR relationships meet the significance threshold of FDR-corrected *P*<0.05 are shown. All statistical tests were two-sided. Source data are provided as a Source Data file.

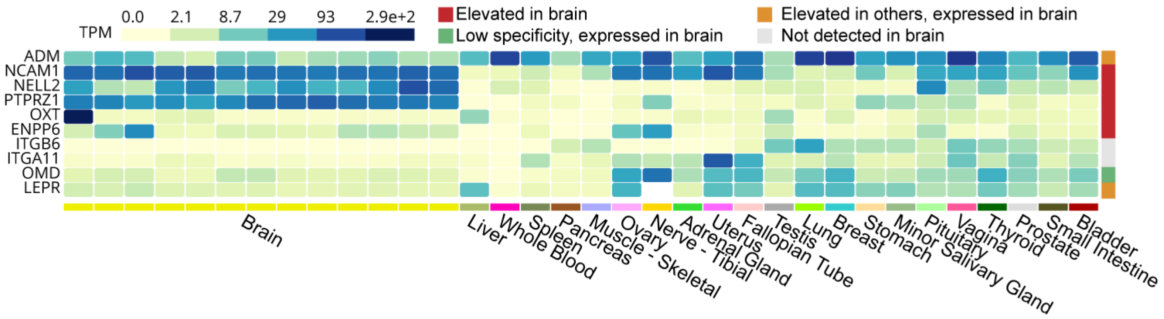

Figure S9 The expression of coding genes across tissues for proteins exhibiting mediation effect. Source data are provided as a Source Data file.

References

1 Sun, B. B. *et al.* Plasma proteomic associations with genetics and health in the UK Biobank. *Nature* **622**, 329-338 (2023).
