# Supplementary material for: Atlas of proteomic signatures of brain structure and its links to brain disorders
Source: Nat Commun. 2025 Jun 2;16:5092. doi: 10.1038/s41467-025-60185-7 (PMC12130460; doi:10.1038/s41467-025-60185-7)
Supplement: Supplementary file 2 — Description of Addtional Supplementary Files [file 41467_2025_60185_MOESM2_ESM.pdf]

Description of Additional Supplementary Files

Supplementary Data S1 - Exclusion criteria for participants

Supplementary Data S2- Field ID for neuroimaging metrics from UK Biobank

Supplementary Data S3- GWAS summary-level data of neurodegenerative and psychiatric disorders.

Supplementary Data S4- Demography of UK Biobank participants used for mediation analysis

Supplementary Data S5- The number of patients in cohorts of different structural categories

Supplementary Data S6- Statistics of significant associations for each measure (FDR-corrected P < 0.05)

Supplementary Data S7- Count of significant associations for each measure (FDR-corrected P < 0.05)

Supplementary Data S8- Statistics of associations for each measure without covariates

Supplementary Data S9- Statistics of associations for each measure without imputation

Supplementary Data S10- Statistics of associations for each measure in subgroup analysis

Supplementary Data S11- Forward MR analysis results (protein to neuroimaing metrics, clumping threshold=5e-6).

Supplementary Data S12- Pleiotropy assessment for the significant results of forward MR analyses (protein to neuroimaging metrics, clumping threshold=5e-6).

Supplementary Data S13- Forward MR analysis results (protein to neuroimaing metrics, clumping threshold=5e-8).

Supplementary Data S14- Pleiotropy assessment for the significant results of forward MR analyses (protein to neuroimaging metrics, clumping threshold=5e-8).

Supplementary Data S15- Reverse MR analysis results (neuroimaing metrics to protein, clumping threshold=5e-6).

Supplementary Data S16- Pleiotropy assessment for the significant results of reverse MR analyses (neuroimaging metrics to protein, clumping threshold=5e-6).

Supplementary Data S17- Reverse MR analysis results (neuroimaing metrics to protein, clumping threshold=5e-8).

Supplementary Data S18- Pleiotropy assessment for the significant results of reverse MR analyses (neuroimaging metrics to protein, clumping threshold=5e-8).

Supplementary Data S19- Forward MR analysis results (protein to disease, clumping threshold=5e-6).

Supplementary Data S20- Pleiotropy assessment for the significant results of forward MR analyses (protein to disease, clumping threshold=5e-6).

Supplementary Data S21- Forward MR analysis results (protein to disease, clumping threshold=5e-8).

Supplementary Data S22- Pleiotropy assessment for the significant results of forward MR analyses (protein to disease, clumping threshold=5e-8).

Supplementary Data S23- Reverse MR analysis results (disease to protein, clumping threshold=5e-6).

Supplementary Data S24- Pleiotropy assessment for the significant results of reverse MR analyses (disease to protein, clumping threshold=5e-6).

Supplementary Data S25- Reverse MR analysis results (disease to protein, clumping threshold=5e-8).

Supplementary Data S26- Pleiotropy assessment for the significant results of reverse MR analyses (disease to protein, clumping threshold=5e-8).

Supplementary Data S27- Statistics for Mediation analysis
