# Supplementary material for: Atlas of proteomic signatures of brain structure and its links to brain disorders
Source: Nat Commun. 2025 Jun 2;16:5092. doi: 10.1038/s41467-025-60185-7 (PMC12130460; doi:10.1038/s41467-025-60185-7)
Supplement: Supplementary file 4 — Reporting Summary [file 41467_2025_60185_MOESM4_ESM.pdf]

nature portfolio

Corresponding author(s): Wei Cheng, Meiyun Wang, Jintai Yu

Last updated by author(s): May 2, 2025

Reporting Summary

Nature Portfolio wishes to improve the reproducibility of the work that we publish. This form provides structure for consistency and transparency in reporting. For further information on Nature Portfolio policies, see our Editorial Policies and the Editorial Policy Checklist.

Statistics

For all statistical analyses, confirm that the following items are present in the figure legend, table legend, main text, or Methods section.

n/a	Confirmed	
☐	☒	The exact sample size (*n*) for each experimental group/condition, given as a discrete number and unit of measurement
☐	☒	A statement on whether measurements were taken from distinct samples or whether the same sample was measured repeatedly
☐	☒	The statistical test(s) used AND whether they are one- or two-sided<br>*Only common tests should be described solely by name; describe more complex techniques in the Methods section.*
☐	☒	A description of all covariates tested
☐	☒	A description of any assumptions or corrections, such as tests of normality and adjustment for multiple comparisons
☐	☒	A full description of the statistical parameters including central tendency (e.g. means) or other basic estimates (e.g. regression coefficient) AND variation (e.g. standard deviation) or associated estimates of uncertainty (e.g. confidence intervals)
☐	☒	For null hypothesis testing, the test statistic (e.g. F, t, r) with confidence intervals, effect sizes, degrees of freedom and P value noted<br>*Give P values as exact values whenever suitable.*
☒	☐	For Bayesian analysis, information on the choice of priors and Markov chain Monte Carlo settings
☒	☐	For hierarchical and complex designs, identification of the appropriate level for tests and full reporting of outcomes
☒	☐	Estimates of effect sizes (e.g. Cohen's *d*, Pearson's *r*), indicating how they were calculated

Our web collection on statistics for biologists contains articles on many of the points above.

Software and code

Policy information about availability of computer code

Data collection	No software was used for collecting data, all raw data to derive Imaging-derived phenotypes (IDPs) were downloaded from the corresponding website
Data analysis	R version v4.2.0 was used for statistical analysis.<br>PLINK v2 was used for processing of UKB genetic data and PLINK v1.9 was used for clumping in MR analysis<br>GTEx v8 online platform was used for analysis of gene expression across tissues<br>FUMA GENE2FUNC module was used for tissue enrichment, scipy was used for enrichment analysis of cell types and UKB panel, WebGestalt online toolkit was used for GO enrichment<br>The TwoSampleMR v0.5.8 R package , RadialMR v1.0 and MR-PRESSO v1.0 were used for MR analyses.<br>SMR v1.3.1 package for SMR analysis<br>lavaan v0.6.17 package in R was used for mediation analysis<br>The code used to run the analyses in this study is publicly available at https://github.com/hitrp/proteomicSignatureOfBrainStructure/

For manuscripts utilizing custom algorithms or software that are central to the research but not yet described in published literature, software must be made available to editors and reviewers. We strongly encourage code deposition in a community repository (e.g. GitHub). See the Nature Portfolio guidelines for submitting code & software for further information.

Data

Policy information about availability of data

All manuscripts must include a data availability statement. This statement should provide the following information, where applicable:

- Accession codes, unique identifiers, or web links for publicly available datasets
- A description of any restrictions on data availability
- For clinical datasets or third party data, please ensure that the statement adheres to our policy

All plasma proteomic, neuroimaging, genomic and mental health phenotype data are publicly available at UK Biobank (http://www.ukbiobank.ac.uk/) and were used in the present study according to application no. 19542 and 202239. The GWAS summary data for disease can be found at the Psychiatric Genomics Consortium (https://www.med.unc.edu/pgc/download-results) and FinnGen (https://www.finngen.fi/fi). The tissue gene expression data are available at GTEx (https://www.gtexportal.org/home/). The GTEx v8 cis-eQTL summary data of brain cortex can be downloaded from https://yanglab.westlake.edu.cn/software/smr/#eQTLsummarydata. All software and analytical methods used in this study are publicly available. More details are available in Methods section.

Research involving human participants, their data, or biological material

Policy information about studies with human participants or human data. See also policy information about sex, gender (identity/presentation), and sexual orientation and race, ethnicity and racism.

Reporting on sex and gender	We used the public data, and the participants were the same as the original data and vary from dataset to dataset. Detailed sample information can be accessed in the corresponding studies.<br>As a study involving human research participants, biological defined sex was considered as a covariate. Moreover, we have also conducted a subgroup analysis of sex in the study design. In UKB participants selected for our study, there's balanced distribution of male and female. Moreover, sex differences were not significant between different categories of brain structural IDPs.
Reporting on race, ethnicity, or other socially relevant groupings	The ethnicity was provided by UK Biobank and we included it as a covariate. The socioeconomic status was measured with the Townsend index.<br>The age at imaging visit, sex, years of education, Townsend index, ethnicity, smoking and drinking status at imaging visit, interval between baseline and imaging visit, scanning site, total intracranial volume are all used as covariates of brain IDPs in our analyses
Population characteristics	The detailed population characteristics of each used data can be found in the corresponding article. Briefly, the population characteristics of the UKB cohort we used are available in Table 1 and Supplementary Table 24
Recruitment	We used the public data, and the participants were the same as the original data and vary from dataset to dataset. Detailed sample information can be accessed in the corresponding original researches.
Ethics oversight	The data we used (e.g. UKB data, GWAS summary, GTEx) are all publicly released, and ethical approval was provided in each study. UKB received approval from the National Information Governance Board for Health and Social Care and the National Health Service North West Centre for Research Ethics Committee (Ref: 11/NW/0382).

Note that full information on the approval of the study protocol must also be provided in the manuscript.

Field-specific reporting

Please select the one below that is the best fit for your research. If you are not sure, read the appropriate sections before making your selection.

☒ Life sciences ☐ Behavioural & social sciences ☐ Ecological, evolutionary & environmental sciences

For a reference copy of the document with all sections, see nature.com/documents/nr-reporting-summary-flat.pdf

Life sciences study design

All studies must disclose on these points even when the disclosure is negative.

Sample size	To enhance study power, the sample size for association were determined by selecting the participants with both plasma proteomic and neuroimaging data. The sample size for GWAS of neuroimaging phenotypes were determined by the largest neuroimaging cohorts available in UKB. This represents the largest data for the same type of research.
Data exclusions	Participants with participants that present obvious brain structural abnormalities were excluded, including all cause dementia, stroke, Parkinson's disease, parkinsonism, Huntington's disease, multiple sclerosis, epilepsy, intracranial injury, cerebrovascular diseases, encephalitis, myelitis, encephalomyelitis, bacterial meningitis, intracranial and intraspinal abscess and granuloma, intracranial and intraspinal phlebitis and thrombophlebitis, cerebral infarction, vascular syndromes of brain in cerebrovascular diseases, other demyelinating diseases of central nervous system, congenital malformations of the nervous system, malignant neoplasm of brain, intracranial laceration and haemorrhage due to birth injury or other degenerative disorders of nervous system.<br>Besides, since the cohort for publicly available genome-wide association study (GWAS) summary of brain IDPs have overlapping with the cohort used for proteomic analysis, we performed GWAS analysis based on 25,576 UKB participants for whom neuroimaging data was available but proteomic data was not.

Replication	No other cohort of this magnitude available for replication at this stage.
Randomization	Not relevant; no experimental procedures were performed as this was a study of Mendel randomization (MR). MR is an epidemiological method that uses genetic variants as instrument to infer the causality between exposure factors and outcomes.
Blinding	Not relevant; there were no experimental groups.

Reporting for specific materials, systems and methods

We require information from authors about some types of materials, experimental systems and methods used in many studies. Here, indicate whether each material, system or method listed is relevant to your study. If you are not sure if a list item applies to your research, read the appropriate section before selecting a response.

Materials & experimental systems

n/a	Involved in the study
☒	☐ Antibodies
☒	☐ Eukaryotic cell lines
☒	☐ Palaeontology and archaeology
☒	☐ Animals and other organisms
☒	☐ Clinical data
☒	☐ Dual use research of concern
☒	☐ Plants

Methods

n/a	Involved in the study
☒	☐ ChIP-seq
☒	☐ Flow cytometry
☐	☒ MRI-based neuroimaging

Plants

Seed stocks	*Report on the source of all seed stocks or other plant material used. If applicable, state the seed stock centre and catalogue number. If plant specimens were collected from the field, describe the collection location, date and sampling procedures.*
Novel plant genotypes	*Describe the methods by which all novel plant genotypes were produced. This includes those generated by transgenic approaches, gene editing, chemical/radiation-based mutagenesis and hybridization. For transgenic lines, describe the transformation method, the number of independent lines analyzed and the generation upon which experiments were performed. For gene-edited lines, describe the editor used, the endogenous sequence targeted for editing, the targeting guide RNA sequence (if applicable) and how the editor was applied.*
Authentication	*Describe any authentication procedures for each seed stock used or novel genotype generated. Describe any experiments used to assess the effect of a mutation and, where applicable, how potential secondary effects (e.g. second site T-DNA insertions, mosiacism, off-target gene editing) were examined.*

Magnetic resonance imaging

Experimental design

Design type	Our analyses include data from structural MRI and diffusion MRI. Please see "Methods" for full details. The information of brain MRI is provided by UK Biobank online refs (https://biobank.ctsu.ox.ac.uk/crystal/crystal/docs/brain_mri.pdf) (Alfaro-Almagro, NeuroImage 2018).
Design specifications	The detailed information of brain MRI scanning is provided by UK Biobank online refs (https://biobank.ctsu.ox.ac.uk/crystal/crystal/docs/brain_mri.pdf) and is full described in the references (Alfaro-Almagro, NeuroImage (2018); Smith et al., Nature Neuroscience (2021); Miller et al., Nature Neuroscience (2016) and Elliott et al., Nature (2018)).
Behavioral performance measures	Behavioral performance in the MRI scanner was not used in this study.

Acquisition

Imaging type(s)	Please see "Methods" for full details. Our analyses include data from T1-weighted structural MRI and diffusion MRI.
Field strength	3T
Sequence & imaging parameters	3D MPRAGE sequence was used for T1-weighted MRI data and SE-EPI sequence was used for diffusion MRI data. The detailed scanning parameters is described in the references (Alfaro-Almagro, NeuroImage (2018); Smith et al., Nature Neuroscience (2021); Miller et al., Nature Neuroscience (2016) and Elliott et al., Nature (2018)), as well as the online refs (https://biobank.ctsu.ox.ac.uk/crystal/crystal/docs/brain_mri.pdf)
Area of acquisition	Siemens' auto-align was used to include the full brain in the imaged field-of-view; this was checked (and corrected if necessary) by the radiographer.

Diffusion MRI ☒ Used ☐ Not used

Parameters: Please see above for information about full details.

Preprocessing

Preprocessing software: The gray matter volume, thickness and area of cortical or subcortical regions were computed with FreeSurfer v6.0.0. The diffusion MRI was processed with FSL

Normalization: The gray matter volume, thickness and area of cortical or subcortical regions from T1w MRI images were measured in subject space, rather than standard space, so the T1w images were not normalized. The diffusion weighted images were normalized with FNIRT-based nonlinear warping in FSL. Specifically, although the Eddy and BEDPOSTx outputs are in the space and resolution of the (GDC-unwarped) native diffusion data space, the nonlinear transformation between subject space and 1mm MNI standard space is used to create tractography results in 1mm standard space. Details processing can be found at https://biobank.ctsu.ox.ac.uk/crystal/crystal/docs/brain_mri.pdf

Normalization template: As mentioned above, the T1w images were in subject space and dMRI images were normalized to FMRIB58_FA_1mm standard space

Noise and artifact removal: Details are available at https://biobank.ctsu.ox.ac.uk/crystal/crystal/docs/brain_mri.pdf or the references (Alfaro-Almagro, NeuroImage (2018); Smith et al., Nature Neuroscience (2021); Miller et al., Nature Neuroscience (2016) and Elliott et al., Nature (2018)). Briefly, the dMRI data is corrected for eddy currents and head motion, and has outlier-slices (individual slices in the 4D) corrected.

Volume censoring: See above (covered previously in full detail in references of Alfaro-Almagro, NeuroImage (2018); Smith et al., Nature Neuroscience (2021); Miller et al., Nature Neuroscience (2016) and Elliott et al., Nature (2018).)

Statistical modeling & inference

Model type and settings: In the association and Mendelian randomization analyses, the mass univariate models were performed for brain IDPs and correct for multiple comparisons. In the mediation analyses, a multivariate modeling was used to construct latent variable using multiple brain IDPs associated with exposures (proteins). More details are available in the "Methods" section.

Effect(s) tested: Neither ANOCA nor factorial designs were used

Specify type of analysis: ☐ Whole brain ☒ ROI-based ☐ Both

Anatomical location(s): Based on the reliable segmentation and meaningful measurement, 272 brain structural IDPs were selected from the original releases, including 218 gray matter IDPs and 54 IDPs of white matter connections. We further classified these IDPs into five regional categories (see Figure 1). Desikan-Killiany (DK) atlas was used for grey matter and 27 major WM tracts in UKB was used for white matter tracts.

Statistic type for inference (See Eklund et al. 2016): ROI-wise statistical inference was made

Correction: FDR correction was used for all analyses, including association analyses, enrichment analyses, Mendelian randomization analyses, where applicable.

Models & analysis

n/a	Involved in the study
☒	☐ Functional and/or effective connectivity
☒	☐ Graph analysis
☐	☒ Multivariate modeling or predictive analysis

Multivariate modeling and predictive analysis: In the association and Mendelian randomization analyses, the univariate models were performed for brain IDPs. In the mediation analyses, a multivariate modeling was used to construct latent variable using multiple brain IDPs associated with exposures (proteins).
